# The Untargeted Phytochemical Profile of Three Meliaceae Species Related to In Vitro Cytotoxicity and Anti-Virulence Activity against MRSA Isolates

**DOI:** 10.3390/molecules27020435

**Published:** 2022-01-10

**Authors:** Leilei Zhang, Maha M. Ismail, Gabriele Rocchetti, Nesrin M. Fayek, Luigi Lucini, Fatema R. Saber

**Affiliations:** 1 Department for Sustainable Food Process, Università Cattolica del Sacro Cuore, Via Emilia Parmense 84, 29122 Piacenza, Italy; leilei.zhang@unicatt.it (L.Z.); luigi.lucini@unicatt.it (L.L.); 2 Microbiology and Immunology Department, Faculty of Pharmacy, Cairo University, Kasr el Aini St., Cairo 11562, Egypt; 3 Pharmacognosy Department, Faculty of Pharmacy, Cairo University, Kasr el Aini st., Cairo 11562, Egypt; nesrin.fayek@pharma.cu.edu.eg (N.M.F.); fatema.saber@pharma.cu.edu.eg (F.R.S.)

**Keywords:** metabolomic profiling, Meliaceae, cytotoxicity, biofilm, MDR, MRSA, staphyloxanthin

## Abstract

Background: A high mortality rate is associated with about 80% of all infections worldwide, mainly due to antimicrobial resistance. Various antimicrobial and cytotoxic activities have been proposed for Meliaceae species. This study aimed to evaluate the in vitro anti-virulence and cytotoxic effect of the leaf extracts of *Aphanamixis polystachya*, *Toona ciliata* and *Melia azedarach* against five MRSA strains and on three cancer cell lines, followed by biological correlation to their encompassed phytoconstituents. Material and Methods: We explored three plants of this family against a panel of Methicillin-resistant *Staphylococcus aureus* (MRSA) strains and several cancer cell lines to select the most promising candidates for further in vivo and preclinical studies. The phytochemical composition was evaluated by UHPLC–QTOF–MS untargeted profiling. Cell viability was assessed by SRB assay. Minimum Inhibitory Concentration was carried out by using the agar micro-dilution technique. Inhibition of biofilm formation and preformed biofilm disruption were assessed spectrophotomertically, according to the Sultan and Nabil method (2019). Results: A total of 279 compounds were putatively annotated to include different phytochemical classes, such as flavonoids (108), limonoids/terpenoids (59), phenolic acids (49) and lower-molecular-weight phenolics (39). *A. polystachya* extract showed the most potent cytotoxic activity against Huh-7, DU-145 and MCF-7 cell lines (IC_50_ = 3, 3.5 and 13.4 µg mL^−1^, respectively), followed by *M. azedarach*, with no effect recorded for *T. ciliata* extract. Furthermore, both *A. polystachya* and *M. azedarach* extracts showed promising anti-virulence and antimicrobial activities, with *A. polystachya* being particularly active against MRSA. These two latter extracts could inhibit and disrupt the biofilm, formed by MRSA, at sub-lethal concentrations. Interestingly, the extracts inhibited hemolysin-α enzyme, thus protecting rabbit RBCs from lysis. *A. polystachya* extract reduced the pigmentation and catalase enzyme activity of tested pigmented strains better than *M. azedarach* at both tested sub-MICs. Consequently, susceptibility of the extract-treated cells to oxidant killing by 200 mM H_2_O_2_ increased, leading to faster killing of the cells within 120 min as compared to the extract-non-treated cells, likely due to the lower antioxidant-scavenging activity of cells exhibiting less staphyloxanthin production. Conclusion: These findings suggested that both *A. polystachya* and *M. azedarach* natural extracts are rich in bioactive compounds, mainly limonoids, phenolics and oxygenated triterpenoids, which can combat MRSA biofilm infections and could be considered as promising sources of therapeutic cytotoxic, antibiofilm and anti-virulence agents.

## 1. Introduction

The Meliaceae family, commonly known as the mahogany family, comprises more than 50 genera and 600 species [1,2], and it is distributed in tropical and subtropical regions [3]. Traditionally, it is used to control pests and produce highly valued timber for construction purposes [1,3]. The three genera *Aphanamixis*, *Toona* and *Melia*, belonging to Meliaceae, have been recently recognized for their cytotoxic activity and other biological activities [4,5,6]. For example, *Aphanamixis* extract was reported to possess anti-inflammatory, antibacterial and antifungal activities [4]. Meanwhile, *Toona* demonstrated antioxidant, anti-infective, antidiarrheal, antidiabetic and leishmanicidal activities [6]. Recently, *Melia* extract was described to have antihemorrhoidal, anthelmintic, antipyretic and antidiabetic activities, with its bark being particularly useful in leprosy, leucoderma and skin diseases [7]. 

The extracts of various parts (leaves, flowers and seeds) of the *Aphanamixis polystachya* (syn. *Amoora rohituka)*, *Toona ciliata* (syn. *Cedrela toona*) and *Melia azedarach* showed antimicrobial activities against *Staphylococcus aureus*, *Staphylococcus epidermidis*, *Pseudomonas areogenes*, *E. coli*, *Bacillus subtilis*, *Salmonella typhi*, *Klebsiella pneumoniae* and *Proteus* spp. [4,8,9,10]. Recently, a *Melia azedarach* leaf extract showed strong inhibition against a panel of dental-biofilm-forming bacteria [11]. Meanwhile, no recent data were found in the scientific literature for the inhibitory potential of the other two species, namely *Aphanamixis polystachya* and *Toona ciliata*, on bacterial biofilms. Overall, a high mortality rate is associated with about 80% of all infections worldwide, mainly due to antimicrobial resistance [12]. Methicillin-resistant *Staphylococcus aureus* (MRSA) is considered one of the MDR bacteria causing life-threatening conditions, including sepsis. In the antibiotic-resistance threat report published in 2019 by the Centre for Disease Control and Prevention (CDC), this bacterium was listed as a severe threat [13]. In addition, this pathogen gained “high-priority” in the global priority antibiotic-resistant-pathogens list released by WHO, for which research and development of novel antibiotics are urgently required [14]. Microbial resistance is associated with bacterial biofilms of the three bacteria most commonly causing wound infections, i.e., *P. aeruginosa*, *Staphylococcus* spp. and *Enterococcus* spp. [15]. These bacterial biofilms, especially *Staphylococcus*, are characterized by their rapid modification of gene expression, thus consequently leading to the alternation of its surface antigens [16,17]. In addition, biofilms increase antibiotic resistance by reducing antibiotic penetration into the cells and altering the growth rate. Moreover, various virulence factors produced by *S. aureus* (hemolysins, proteases, nucleases, the antioxidant staphyloxanthin pigment and others) play an important role in bacterial pathogenesis and resistance [18]. On the other hand, malignant tumors are considered the second leading cause of death globally, after ischemic heart disease and stroke, accounting for an estimated 9.6 million deaths, or one in six deaths, in 2018 [19]. The most common types of cancer in men are prostate, liver, lung, colorectal and stomach cancer. Meanwhile, among women, breast, colorectal, cervical and thyroid cancer are the most predominant [19]. 

Starting from this scenario, the quest for efficient alternatives to classical control agents has gained primary attention. In this regard, various cytotoxic activities have been proposed for Meliaceae, both in vitro and in vivo. Among others, promising properties have been reported for *A. polystachya* [20], *T. ciliata* [21,22] and *M. azedarach* [5]. The cytotoxic compounds of *A. polystachya* and *M. azedarach* are supposed to be flavonoids, steroids, triterpenoids (such as tirucallane), diterpenoids, limonoids and organic acids [20,23]. Therefore, this study aimed to explore the phytochemical profile and evaluate the in vitro antibacterial and anti-virulence potential of the leaf extracts of the three meliaceous plants against a panel of five MRSA strains. A second aim was to evaluate the cytotoxic activity of the selected leaf extracts and further to correlate the encompassed phytoconstituents, as revealed by UHPLC–QTOF–MS untargeted analysis, with biological activities.

## 2. Results and Discussion

### 2.1. Phytochemical Profiling of Meliaceae Extracts Using UHPLC–QTOF–MS

In this work, untargeted metabolomics was used to comprehensively investigate the three Meliaceae species (i.e., *Toona ciliata*, *Aphanamixis polystachya* and *Melia azedarach*). Overall, the untargeted approach enabled the putative annotation of 279 compounds (excluding isobaric compounds), and the resulting dataset is reported in the Supplementary Materials, together with the relative abundance and mass spectra of each compound. The detected phytochemicals belonged to different chemical classes, such as flavonoids (108), limonoids/terpenoids (59), phenolic acids (49), lower-molecular-weight (LMW) phenolics (39), lignans (17) and stilbenes (7). In particular, anthocyanins (pigmented flavonoids) were primarily identified in *T. ciliata*, up to 104.16 mg Eq./g dry matter (Table 1) and mainly represented by cyanidin, pelargonidin, malvidin, peonidin, petunidin, delphinidin and their glycosides. Moreover, the metabolic profile of *T. ciliata* extract was dominated by LMW phenolics and flavonoids, followed by limonoids. Nevertheless, both *M. azedarach* and *A. polystachya* extracts (dichloromethane: methanol extracts) were abundant in limonoids (as the major phytoconstituents; 420.63 and 433.17 mg Eq./g dry matter, respectively). Similarly, Jarvis et al. [24] reported the enrichment of the dichloromethane fraction of *Azadirachta indica* seeds with the oxygenated triterpenoids. Noteworthy, 11*beta*-hydroxycneorin G, a previously isolated compound from *Cedrela sinesis*, was identified as a major limonoid in the three investigated Meliaceae species. Additionally, 1-*O*-deacetylohchinolide A, ohchnolide B, 1-*O*-Deacetyl-1-*O*-tigloylohchinolide B were also common limonoids detected in high abundance in the three genera.

Regarding *M. azedarach* phytochemical profile, the major limonoids detected included nimbolide, nimonol, ohchnolide B, limonol and amoorinin. The sesquiterpenoids, 6*beta*,7*beta*-epoxyguai-4-en-3-one and 6*beta*,7*beta*-epoxy-4*beta*,5-dihydroxyguaiane were found in *A. polystachya* and *M. azedarach* leaves. They were previously characterized by Chowdhury et al. [25] from *A. polystachya* stem bark. According to our results, amooramides A, E, D and F were enriched and may represent potential markers in *Aphanamixis* metabolome. At the same time, amooramides C, B and G were also common between *Toona* and *Melia* leaf extracts. Noteworthy, these compounds were previously characterized in *Amoora tsangii* [26]. 

On the other hand, 17 lignans were identified in *Toona*, *Aphanamixis* and *Melia* extracts, exemplified by arctigenin, 7-hydroxy secoisolariciresinol, cyclolariciresinol and secoisolariciresinol. However, they were present in higher abundance in *Aphanamixis* leaves’ extract (188.56 mg Eq./g dry matter). This agrees with previous reports characterizing lignans from the Meliaceae family [27,28]. Regarding other phenolics identified in the investigated Meliaceae species; coumarins, stilbenes, hydroxycinnamic acids, tyrosol equivalents and alkylphenols, were also identified. Hydroxycinnamates represented by *p*-coumaric acid and verbascoside are dominant in *Toona*, while caffeoylquinic, coumaroylquinic and isoferulic acids were the chief phenolic acids identified in *M. azedarach* extract. Protocatechuic acid 4-*O*-glucoside, syringic acid, *p*-coumaric acid 4-*O*-glucoside were abundant in *A. polystachya*.

### 2.2. Multivariate Statistical Discrimination Analysis of Meliaceae Species

**Figure 1:**
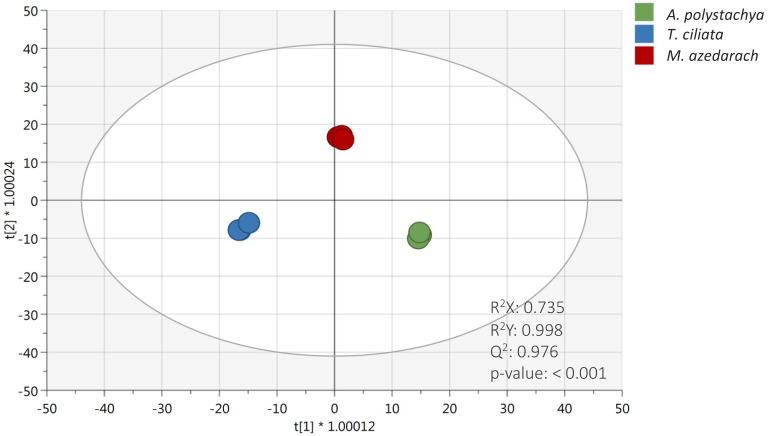
OPLS-DA score plot considering the different extracts of Meliaceae species under investigation. The accuracy parameters of the prediction model are also provided in the score plot.

To discriminate the three Meliaceae species based on their metabolic profile, an unsupervised hierarchical clustering analysis (HCA) was carried out. The fold-change-based heat map resulting from HCA showed clear discrimination of three species (Supplementary Materials Figure S1). Accordingly, the heat map showed two main groups: the first cluster was represented by the phytochemical profile of *Toona ciliata*, while the second cluster included both *Aphanamixis polystachya* and *Melia azedarach*, thus suggesting a higher hierarchical similarity between these latter. Interestingly, the heat map clearly showed that *T. ciliata* extract was characterized by a set of compounds exclusively abundant when compared to *A. polystachya* and *M. azedarach*. These differences could be attributed to the high concentration of anthocyanins found in this species, as shown in Table 1. However, both *A. polystachya* and *M. azedarach* extracts showed an up-accumulation of different compounds, likely including lignans, LMW phenolics and phenolic acids that were the most abundant compared to *T. ciliata* extract. As the next step, to better describe the differences in phytochemical profiles detected, a supervised OPLS-DA approach was used, and the resulting score plot is reported in Figure 1. The robustness of the OPLS-DA model showed more than acceptable accuracy parameters; indeed, the goodness-of-fit R^2^X and R^2^Y were equal to 0.735 and 0.998, respectively, while the goodness-of-prediction (Q^2^Y) was equal to 0.976. Moreover, the output of permutation test cross-validation (N = 200; see Supplementary Materials) revealed no overfitting in the model built. This approach proved to be particularly effective for detecting discriminant compounds among samples. Indeed, as shown in Figure 1, the OLPS-DA model resulted in a clear separation of *T. ciliata* from *A. polystachya* and *M. azedarach* on the first latent vector t [1], while the second latent vector t [2] was able to discriminate *M. azedarach* from *A. polystachya* and *T. ciliate* extracts. The variables’ importance in projection (VIP) of the OPLS-DA model was then evaluated according to a prediction score > 1. The comprehensive list of those potential VIP marker compounds, together with their VIP score and LogFC values obtained by comparing the significant metabolites of the different species (e.g., *T. ciliata* vs. *A. polystachya*, *M. azedarach* vs. *A. polystachya,* and *M. azedarach* vs. *T. ciliata*), can be found in the Supplementary Materials. This approach allowed us to identify 164 discriminant compounds among the studied species. Among these potential markers, limonoids, such as nimonol, 1-cinnamoyl-3,11-dihydroxymeliacarpin, amooramide B, cedrelone and azadironolide, showed the higher VIP score (ranging from 1.19 to 1.15), followed by flavonoids, such as quercetin 3-*O*-xylosyl-rutinoside, 6″-*O*-malonylgenistin, cyanidin 3-*O*-glucosyl-rutinoside and dihydromyricetin 3-*O*-rhamnoside. Moreover, both hydroxycinnamic acids and alkylphenols (such as *p*-coumaroylquinic acid and 5-pentadecylresorcinol, respectively) were highlighted as the most discriminant compounds for the three Meliaceae species. In line with our findings, cedrelone was previously reported as the limonoid mainly characterizing and discriminating *T. ciliata* species [29].

### 2.3. In Vitro Cytotoxic Activity of Meliaceae Extracts

**Figure 2:**
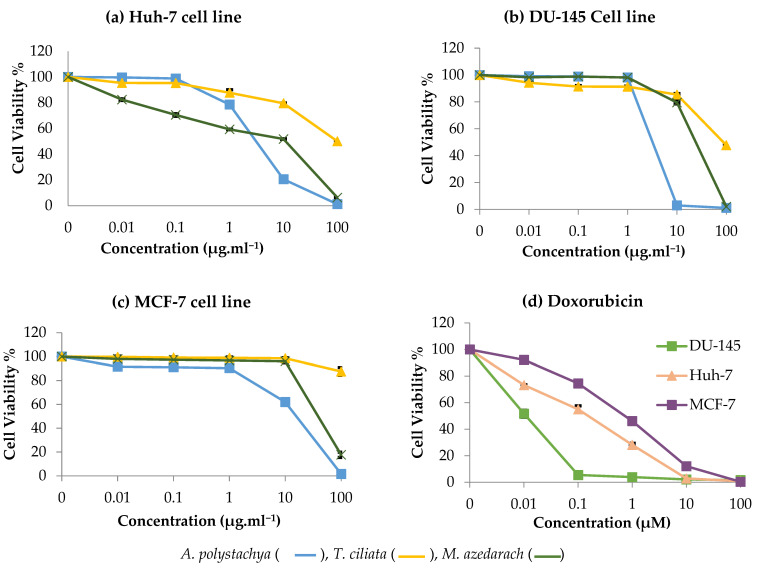
In vitro cytotoxic activity of *A. polystachya*, *T. ciliata* and *M. azedarach* extracts, using Sulforhodamine B Assay (SRB assay) against 3 cancer cell lines: (**a**) Huh-7 (human hepatocellular carcinoma cell), (**b**) DU-145 (human prostate carcinoma cell) and (**c**) MCF-7 (human breast adenocarcinoma cell). (**d**) In vitro cytotoxic activity of Doxorubicin used as drug reference. Data are represented as mean ± SD (*n* = 3).

The leaf extracts of *T. ciliata*, *A. polystachya* and *M. azedarach* were evaluated for their cytotoxic potential against three cancer cell lines: Huh-7 (human hepatocellular carcinoma cell), DU-145 (human prostate carcinoma cell) and MCF-7 (human breast adenocarcinoma cell). The results showed that both *M. azedarach* and *A. polystachya* extracts are powerful cytotoxic agents against Huh-7 liver cancer cells in contrast to that of *T. ciliata* (IC_50_ = 2.3, 3 and >100 µg mL^−1^, respectively) (Figure 2). Additionally, *A. polystachya* extract displayed stronger activity against DU-145 and MCF-7 cell lines than *M. azedarach* (IC_50_ = 3.5 and 13.4 µg mL^−1^ versus 18.3 and 47 µg mL^−1^, respectively). On the other hand, *T. ciliata* extract was inactive as a cytotoxic agent against the three tested cell lines. However, the observed in vitro cytotoxicity of *M. azedarach* and *A. polystachya* extracts was inferior to that of Doxorubicin; Doxorubicin showed IC_50_ of 0.01, 0.11 and 0.63 µM (equivalent to 0.0054, 0.059 and 0.342 µg mL^−1^) against DU-145, Huh-7 and MCF-7 cell lines. A previous study reported a moderate cytotoxic effect of toonaciliatavarin D and toonaciliatavarin E isolated from *T. ciliata* against KB (oral epithelial), K562 (leukemia) and SMMC-7721 (hepatocellular) cancer cell lines. At the same time, the other six limonoids were inactive (IC_50_ > 50 µM). Noticeably, all compounds were inactive against MCF-7 cell lines [22]. The superiority of the extract of *M. azedarach* to *T. ciliata* as cytotoxic agent against the tested cell lines could also be attributed to encompassing less of the known cytotoxic limonoids, such as nimbolide and nimonol [30,31].

The extraction method could also influence the entailed phytoconstituents and hence the observed bioactivity. A bioactive fraction from the aqueous extract of *Toona sinensis* induced apoptosis in ovarian cancer cell lines (SKOV3); remarkably, other fractions were inactive (IC_50_ > 100 µg mL^−1^). This contrasts with our results, where the limonoid-rich fraction prepared from *T. ciliata*, a closely related species, was assessed in our study and exhibited no cytotoxic potential. Mechanistically, the cytotoxic activity of certain limonoids, such as nimbolide, azadiradione and benzoylgedunin derivatives, involves apoptotic effects (both early and late) against HL60 leukemia cell lines [30]. Regarding the cytotoxic activity of *A. polystachya* extract, previous reports highlighted the presence of potent cytotoxic limonoids in *A. polystachya* as Nornemoralisins A and B [20]. However, other researchers reported that phytochemicals from other *Aphanamixis* species showed moderate to minimal activities as cytotoxic [32]. Structurally, the increased conjugation in the limonoids of *Aphanamixis* has a great impact on its cytotoxic potential [32].

### 2.4. Antimicrobial Testing

#### 2.4.1. Determination of the MIC of the Tested Extracts

The Meliaceae family is known for its antimicrobial activity, with different genera of this family showing significant antibacterial and antifungal activities against a wide range of pathogens, including MRSA [3]. The antibacterial activity of the polar and non-polar seed extracts of *M. azedarach* and stem-bark extracts of *A. rohituka* against a wide range of Gram-positive and Gram-negative pathogenic bacteria was also reported [33,34]. In this work, *A. polystachya* extract showed significantly greater antimicrobial activity compared to *M. azedarach* extract (i.e., 2.42 and 6.7 mg mL^−1^, respectively; level of significance is 99.99%; and *p*-value < 0.0001). Both tested extracts showed antimicrobial activity that is inferior to the activity recorded for vancomycin (Supplementary Materials Figure S2).

In this study, in both *A. polystachya* and *M. azedarach* extracts, limonoids were detected as the major phytoconstituents; this is in agreement with Lu et al. [35], who reported the isolation of 15 limonoids from the ethanolic extract of the dry seeds of neem. Among these isolated compounds, the antibacterial activity was observed against both Gram-positive and Gram-negative isolates. Another study conducted by Rahman [36] reported the isolation of swietenolide and 2-hydroxy-3-*O*-tigloylswietenolide from *Swietenia mahagoni* (family Meliaceae), showing activity against eight MDR clinical isolates, including *S. aureus*. However, their antibacterial activity was inferior to that of vancomycin, which was included as an antibiotic standard that agrees with our study. The observed antimicrobial activity is mainly attributable to their enrichment with limonoids, in addition to other phenolics which act via disruption of the bacterial membrane, leading to the loss of its selective permeability and subsequent killing of pathogens. It is noteworthy that nimbolide, a major constituent detected in the LC–MS profile of *M. azedarach*, exhibited cell membrane disruption and biofilm inhibition of multidrug-resistant MRSA, and this effect was dose-dependent [37].

#### 2.4.2. Anti-Virulent Activity of the Tested Extracts

Virulence factors produced by MRSA allow its survival inside the host and evasion of the immune system; these factors include hemolysins (α, β, γ and δ) nuclease, protease and other enzymes [38]. Anti-virulence therapy is an attractive alternative strategy to limit microbial resistance rather than killing a pathogen. This includes inhibition of biofilm formation, disruption of preformed biofilm, inhibition of certain bacterial enzymes and a reduction of bacterial pigmentation [39].

**Figure 3:**
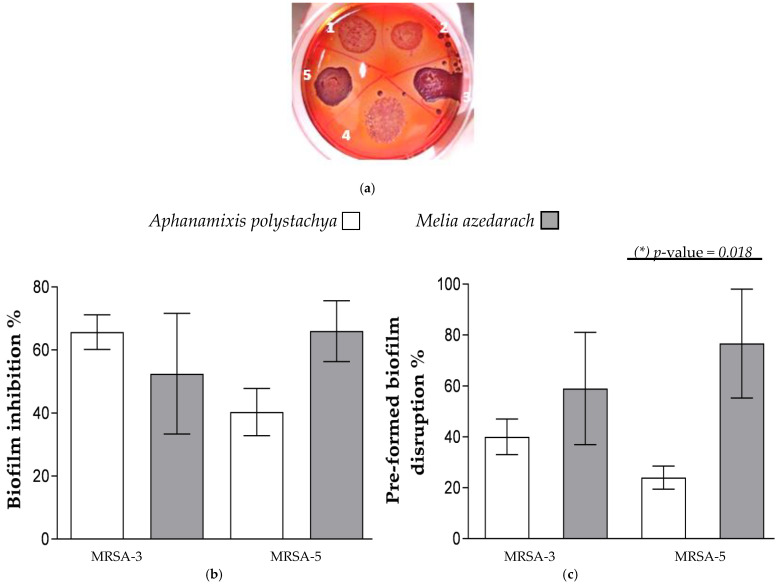
(**a**) Congo red agar medium showing black colonies production with strains MRSA-3 and MRSA-5 only, indicating positive slime production. Pink colonies indicate negative slime production. (**b**) Percentage inhibition of biofilm formation and (**c**) percentage disruption of preformed biofilm formed by MRSA-3 and -5 strains by *Aphanamixis polystachya* and *Melia azedarach* extracts at concentration of 0.125 mg mL^−1^. Data represent the means of percentage biofilm inhibition/disruption ± SD, *n* = 3 (one-way ANOVA, followed by Tukey’s multiple comparisons, *p*-value < 0.05). (*) Indicates presence of a statistically significant difference between columns.

At first, Inhibition of Biofilm Formation and Preformed Biofilm Disruption by the Tested Extracts were examined. Biofilm is an important virulence-associated trait in pathogenic bacteria that allows for the colonization and adhesion of bacteria to host tissue for the establishment of disease [40]. The qualitative and quantitative screening of biofilm formation (BF) by the tested MRSA strains showed that only two strains, MRSA-3 and MRSA-5, are strong biofilm-producing strains (Figure 3a) while others are non-biofilm-forming. Both strains showed black colonies on CRA medium and BF ≥ 0.300 (0.64 ± 0.2 and 0.49 ± 0.1 for MRSA-3 and MRSA-5, respectively). The effects of the extracts on biofilm inhibition and preformed biofilm disruption were tested at concentrations ranging from 0.125 to 0.07 mg mL^−1^. Moreover, these concentrations showed to be stable when mixed with the medium, considering discoloration or precipitation; thus, they did not interfere with biofilm visualization and quantitation. Both extracts of *A. polystachya* and *M. azedarach* can inhibit and disrupt the biofilm formed by MRSA-3 and -5. No statistically significant difference was observed between the anti-biofilm activities of both extracts. It was noticed that only the preformed biofilm disruption ability of *M. azedarach* extract is statistically higher than that caused by *A. polystachya* against MRSA-5 strain (Figure 3b,c).

Phytochemicals have demonstrated different modes of antimicrobial potential [41,42]. Bacterial biofilms are implicated in about 80% of microbial infectious diseases [43]. They consist of exopolysaccharides that conserve the bacteria from being attacked by antimicrobials and are more resistant to environmental conditions up to 1000 times than their planktonic forms [44].

In this study, the major detected chemical class was limonoids, followed by lignans and phenolics. Limonoids were reported to inhibit bacterial biofilm formation via suppressing the signaling of auto-inducer peptides (AIPs) (the quorum sensing (QS) mediators in Gram-positive bacteria). The AIPs play a critical role in biofilm formation and expression of virulence factors [45]. Limonoids could also suppress the production of intercellular adhesin polysaccharide of MRSA, leading to the suppression of cell-to-cell adhesion required for biofilm formation and planktonic aggregation [46]. Lignans are considered among the major chemical classes detected in this work, especially in *A. polystachya*. Lignans are reported for their ability to reduce the production of exopolysaccharides important for biofilm formation, in addition to downregulation of QS gene expression in *P. aeruginosa*. As a consequence, the production of virulence factors and enzymes is suppressed [47]. Phenolics could kill pathogens and inhibit biofilm via different mechanisms: disruption of the bacterial membrane (with subsequent leakage of cellular content) and inhibition of cell–cell adhesion, inhibition of extracellular matrix formation and inhibition of Staphylococcal protein A, thus preventing bacterial adhesion and attachment required for biofilm production [45]. A possible interaction between compounds of these chemical classes has led to the observed anti-virulence effect of the extracts.

Nevertheless, a further in vivo study is recommended for the major isolated compounds from the three Meliaceae species for further characterization and confirmation of the suggested activity.

**Figure 4:**
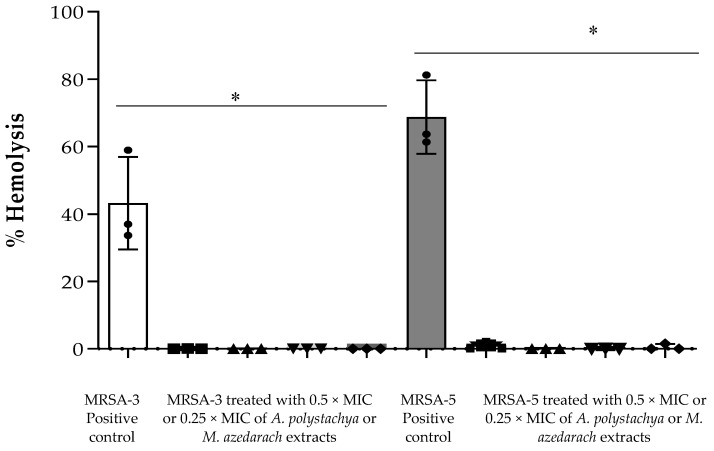
Percentage of hemolysis activity by hemolysin-α (Hla) of the culture supernatants of MRSA-3 or MRSA-5 treated or untreated with either *Aphanamixis polystachya* or *Melia azedarach* extracts. The symbols ■ and ▲ represent the cells treated with 0.5 × MIC and 0.25 × MIC of *Aphanamixis polystachya* extract, respectively; ▼ and ◆ represent the cells treated with 0.5 × MIC and 0.25 × MIC of *Melia azedarach* extract, respectively. (*) Indicates presence of a statistically significant difference between columns (one-way ANOVA, followed by Tukey’s multiple comparisons, *p*-value < 0.0001). Data represent the means ± SD, *n* = 3.

Then, Effect of the Extracts on α-Hemolysin Activity (Hla) was investigated. Hla is one of the most important virulence factors produced by *S. aureus*, inducing damages and lysis in many types of cells, including pneumocytes. Thus, Hla is considered an important target in the anti-virulence therapy of *S. aureus,* which helps its pathogenesis in the invaded cells [48,49]. In this study, only MRSA-3 and -5 strains showed α-hemolysin activity against rabbit RBCs. Both *A. polystachya* and *M. azedarach*, at the tested concentrations (0.5 × and 0.25 × MIC), showed complete inhibition of Hla activity (Figure 4). No previous studies on the inhibition of Hla by members of Meliaceae family have been reported. In general, many studies have investigated the role of plant-based products as inhibitors for Hla. Ping et al. (2018) reported a natural chromone compound (Prim-*O*-glucosylcimifugin (POG)) that could inhibit the secretion of Hla in *S. aureus* strain USA300 at the sub-MIC concentrations. Another study performed by Wang and coworkers [50] identified myricetin as effectively inhibiting the oligomerization of Hla, thus attenuating its cell lysis activity at sub-lethal concentrations. Natural products were reported to inhibit the formation of this exotoxin as represented by eugenol and hydroxytyrosol [51]. To the best of our knowledge, this is the first report on the inhibitory effect of *A. polystachya* and *M. azedarach* against Hla.

Staphyloxanthin, a golden-yellow pigment that possesses antioxidant activity, is another virulence factor of *S. aureus* that is involved in protecting bacteria from reactive oxygen species (ROS) and enhancing the resistance of innate immune response represented by the host neutrophils [52]. Among the tested MRSA strains, only two (MRSA-3 and -5) showed golden pigmentation, whereas other MRSA strains were white. The effect on pigment production by the action of *A. polystachya* was more noticeable than that of M. azedarach for both tested concentrations (Supplementary Materials Figure S2). As mentioned above, the observed reduction of pigmentation and hemolysis activity could be a result of suppression of QS signaling molecules in *S. aureus* (AIPs) and a possible downregulation of virulence-associated genes reported for the major detected phytoconstituents in this study.

#### 2.4.3. Effect on Catalase Enzyme and Susceptibility to Killing by H_2_O_2_

**Figure 5:**
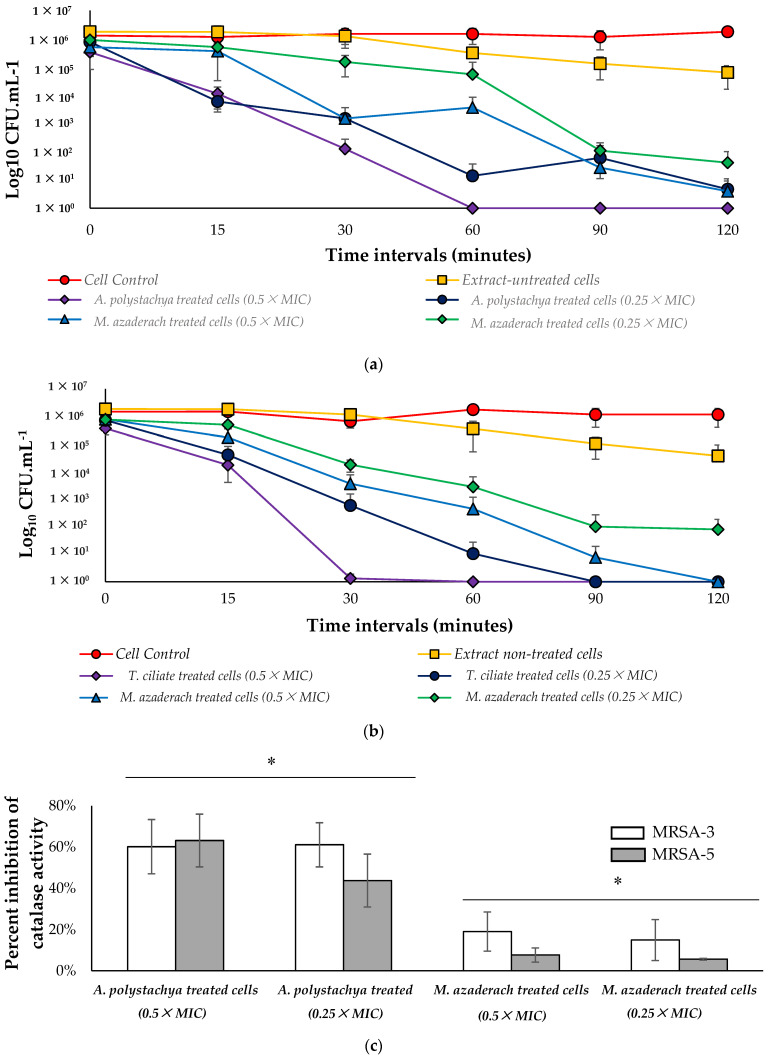
(**a**,**b**) Kill kinetics of MRSA-3 and MRSA-5 strains, respectively, exposed to 200 mM H_2_O_2_ and pretreated with either *Aphanamixis polystachya* or *Melia azedarach* extracts within 120 min. Data represent the means of the viable colonies recovered at each time point ± SD, *n* = 3. (**c**) Inhibition of catalase enzyme activity in cells pretreated with either *Aphanamixis polystachya* or *Melia azedarach* extracts. Data represent the means of the height of the observed O_2_ froth that remained constant for 15 min ± SD, *n* = 3. One-way ANOVA, followed by Tukey’s multiple comparisons, *p*-value < 0.0001). (*) Indicates presence of a statistically significant difference between columns.

In this study, we examined the time-kill kinetics of MRSA-3 and MRSA-5 cells pretreated with a sub-MIC concentration of either *A. polystachya* or *M. azedarach* extracts exposed to 200 mM H_2_O_2_. *A. polystachya* extract increases the susceptibility of both MRSA-3 and -5 strains to killing by H_2_O_2_; at 0.5 × MIC, these strains were killed within 60 and 30 min, respectively, while at 0.25 × MIC, more extended time was needed to achieve complete killing, 120 and 90 min, respectively. At 0.5 × MIC of *M. azedarach* extract, both tested strains were killed within 120 min, while 0.25 × MIC resulted only in 4 log cycle reduction, not complete killing, within 120 min. Untreated cells subjected to 200 mM H_2_O_2_ exhibited only 2 log cycle reduction (Figure 5a), indicating the ability of the tested extracts to increase the sensitivity of the cells to killing by H_2_O_2_ within 2 h in a dose-dependent manner. No reduction in the viable count was observed in the negative control cells, which were not treated with extract and not subjected to 200 mM H_2_O_2_. Moreover, both extracts showed inhibition of catalase enzyme activity. In both tested MRSA-3 and MRSA-5 strains, *A. polystachya* extract showed a higher statistically significant inhibition effect on catalase enzyme than that of *M. azedarach* at both tested concentrations (Figure 5b). This finding correlates with the observed higher activity of *A. polystachya* extract in increasing the susceptibility of the treated cells to be killed by 200 mM H_2_O_2_ faster than *M. azedarach* extract. The disruption of pigment production by *S. aureus* subsequently increases its susceptibility to oxidants [53]. Catalase is produced in the bacterial cell to decrease the harm associated with produced ROS inside bacterial cells; this triggered the search for antimicrobials affecting the bacterial redox system [54]. The observed effects of both *Aphanamixis* and *Melia* extracts to increase the bacterial sensitivity to killing by H_2_O_2_, and the inhibition of catalase enzyme suggest the pro-oxidant activity of these extracts at the tested sub-lethal concentrations. Pro-oxidants can induce structural alterations in microorganisms and thus delay their growth [55]. The increased susceptibility of *S. aureus* to oxidants by botanicals has been investigated. A mutant *S. aureus* with impaired pigment biosynthesis was observed to be more vulnerable to oxidant killing and was less pathogenic to mice in a subcutaneous abscess model [56]. Thus, the ability of both *M. azedarach* and *A. polystachya* extracts to reduce pigmentation of the tested *S. aureus* strains, which increased their susceptibility to H_2_O_2_ killing, agrees with previous studies from other botanical sources [57].

### 2.5. Pearson’s Correlations between Phytochemical Profile and Biological Activity

Pearson’s correlation coefficients were finally evaluated to investigate different degrees of correlations between the main classes of phytochemicals and the various biological activities under investigation, such as cytotoxic activity against Huh-7, MCF-7 and DU-145, as well as antimicrobial or antibiofilm formation of MRSA strains. A summarizing correlation table is provided in the Supplementary Materials.

Overall, lignans and limonoids were the classes of compounds that were most related to the biological activities, and this was true mainly for the extracts of both *M. azedarach* and *A. polystachya*. Indeed, the cytotoxic activity against three cancerous cell lines exhibited a significant negative correlation with these phytochemical classes. In detail, plant extracts containing high amounts of lignans were negatively correlated with the viability of MCF-7 (r = −0.87; *p* < 0.01) and DU-145 (r = −0.73; *p* < 0.05) cell lines, proving to be the class primarily responsible for the negative correlation. Furthermore, plant extracts that were enriched in limonoids resulted in being the greater inhibitor of the three cancer cell lines, showing strong negative correlations ranging between −0.93 and −0.99 (*p* < 0.001). Recent studies have proved that both market drugs containing lignans and lignan-rich fractions have an anticancer effect [2]. For example, the Egyptian flaxseed, especially Giza-9, and its dietary formulations are enriched with lignans and exerted an in vitro and in vivo anticancer effect on a human breast cancer cell line and in mice bearing tumors [58]. Nano-formulated nimbolide, is characterized by its sustained release for more than 6 days in PBS (pH 7.4) and also exerted enhanced cytotoxicity about two- to three-fold in both breast and pancreatic cancer cell lines compared with free nimbolide [59].

Breast cancer is common among the global women population, and the conventional procedure for its treatments includes surgical removal of the malignant tissue, ionizing radiation and chemotherapy, causing several side effects. Compounds derived from plant extracts showed to be the next alternative medicine against cancer, with reduced side effects and the ability to boost up the immune system to fight against metastatic cells. In compliance, the cytotoxicity capacity of *A. rohituka* leaves extract against human breast adenocarcinoma (MCF-7), triple-negative human breast cancer (MDA-MB-231), mice undifferentiated carcinoma (EAC) and mice fibroblast (L929) cell lines was reported by different authors owed to the presence of alkaloids, flavonoids, steroids, tannins, saponins and terpenoids [60,61,62].

The same result was observed for the antimicrobial activity against the five MRSA strains. In this regard, lignans and limonoids showed a strong negative correlation with antimicrobial activity (on average r = −0.66; *p* < 0.05 and r = −0.99; *p* < 0.01, respectively). These findings suggested that an increased quantity of these compounds resulted in a low extract concentration needed to inhibit the growth of cancer cells or MRSA strains. Moreover, we observed a dose–response effect between these classes of compounds and the time needed to kill MRSA strains, as demonstrated by the time-kill assay. Chowdhury et al. [33] studied the antiviral and antibacterial activity of the limonoid rohitukine, which is found in *A. rohituka* extracts. In addition, Yuan et al. [63] showed the same activity of four limonoids (i.e., 3-deacetylkhivorin, 1-deacetylkhivorin, swietmanin B and 3-beta-acetoxy-1-oxo-methylmeliacate) against *Staphylococcus aureus*, *Pseudomonas aeruginosa* and two other clinically isolated bacterial (MRSA) strains. In the current study, the antibiofilm activity was evaluated on the strains MRSA-3 and -5, expressed in terms of inhibition capacity of biofilm formation and disruption of preformed biofilm. Interestingly, the strong positive correlation reported by limonoids in inhibiting biofilm formation on MRSA-3 and -5 strains (r = 0.98 and 0.87; *p* < 0.01, respectively). The wide spectrum of action of limonoids was extended also in the disruption capacity of preformed biofilm (r = 0.86 and 0.68, respectively). Moreover, phenolic acids identified in our Meliaceae species provided a significant capacity to regulate the biofilm formation both in early and preformed phases for MRSA 5. In accordance with our findings, several authors reported the efficacy of phenolic acids in antibiofilm formation [64,65]. In particular, Walker et al. [66] reported the capacity of rosmarinic acid, a natural phenolic acid, to prevent *Pseudomonas aeruginosa* biofilm formation. Other phenolic compounds were also found to be capable of inhibiting QS by using different approaches, such as catechin [67] and eugenol [68].

## 3. Materials and Methods

### 3.1. Plant Material

Leaves of *Toona ciliata* M. Roem., *Aphanamixis polystachya* (Wall.) R. Parker and *Melia azedarach* L. were collected at the flowering stage on April/May 2019 from Orman Botanical Garden, Dokki, Giza, Egypt. The identity of the plant material was confirmed by staff members of the herbarium at Orman Botanical Garden. Voucher specimens (12.04.2019I, 13.05.2019I and 13.05.2019II, respectively) were deposited at Pharmacognosy Department, Faculty of Pharmacy, Cairo University, Egypt.

### 3.2. Preparation of Plant Extracts

The powdered leaves of the Meliaceae species under investigation (300 g each) were extracted in a Soxhlet apparatus (Glassco, India), using dichloromethane–methanol (80:20, *v*/*v*). The extracts were then evaporated till dryness at 50 °C, under reduced pressure, using a rotary evaporator. The dried extracts were subsequently kept in a dry and dark place at 10 °C until further analyses.

### 3.3. In Vitro Cytotoxic Assay

Three cell lines, namely Huh-7 (human hepatocellular carcinoma cell), DU-145 (human prostate carcinoma cell) and MCF-7 (human breast adenocarcinoma cell), were obtained from Nawah Scientific Inc. (Almokattam, Cairo, Egypt). Cells were maintained in DMEM medium supplemented with 100 mg mL^−1^ of streptomycin, 100 units mL^−1^ of penicillin and 10% of heat-inactivated fetal bovine serum in humidified 5% (*v*/*v*) CO_2_ atmosphere at 37 °C. Cell viability was assessed by Sulforhodamine B Assay (SRB) assay [69]. Aliquots of 100 μL cell suspension (5 × 10^3^ cells) were placed in 96-well flat-bottom tissue-culture plates and incubated in complete media for 24 h. Cells were treated with another aliquot of 100 μL media containing the 3 plant extracts at various concentrations (0.01, 0.1, 1, 10 and 100 μg/mL) and Doxorubicin (0.01, 0.1, 1, 10 and 100 μM). After 72 h of drug exposure, cells were fixed by replacing media with 150 μL of 10% TCA and incubated at 4 °C for 1 h. The TCA solution was removed, and the cells were washed 5 times with distilled water. Aliquots of 70 μL SRB solution (0.4% *w*/*v*) were added and incubated in a dark place at room temperature for 10 min. Plates were washed 3 times with 1% acetic acid and allowed to air-dry overnight. Then 150 μL of 10 mM tris was added to dissolve the protein-bound SRB stain and the absorbance measured at 540 nm, using a BMG LABTECH^®^- FLUOstar Omega microplate reader (Ortenberg, Germany). Results are provided in Figure 2.

### 3.4. Microbiological Testing

#### 3.4.1. Bacterial Isolates and Culture Conditions

Five strains of methicillin-resistant *Staphylococcus aureus* (MRSA) isolated from intensive-care-unit patients of two Egyptian hospitals were used in this study [70]. The strains were grown aerobically on Trypticase soy broth/agar (TSB/TSA) (Oxoid, UK), either under shaking at 180 rpm or static conditions, at 37 °C for 24 h. The strains were preserved and maintained by cryopreservation. All microbiological-related testing and assays were performed in triplicate, otherwise specified.

#### 3.4.2. Determination of the Minimum Inhibitory Concentration (MIC) of the Plant Extracts by Agar Dilution Technique

MIC was determined according to the method described by (Jenkins and Schuetz, 2012). In brief, the dry extracts were dissolved in absolute alcohol to a final concentration of 250 mg mL^−1^. Different concentrations of each extract solution were prepared in molten Muller Hinton agar [71] medium at 55–60 °C, and then MHA–extract mixture was poured into 6 cm Petri dishes and left to solidify. Then 10 µL of 10^7^ CFU mL^−1^ bacterial suspension was spotted on the surface of the solidified MHA–extract mixture. Negative and positive controls were included (MHA–absolute alcohol without bacteria and MHA–absolute alcohol surface inoculated with bacteria, respectively). Plates were incubated at 37 °C for 24 h. MHA plates with different concentrations of vancomycin, as a reference antibiotic, were included. MIC was recorded as the lowest concentration of extract showing no visible growth.

### 3.5. Screening of Biofilm Formation by the Tested Strains

#### 3.5.1. Qualitative Screening Using Congo Red Agar (CRA) Medium

Slime production is an important phenotype associated with biofilm formation [72]. A total of 10 µL of 10^7^ CFU mL^−1^ bacterial suspension was spotted on the surface of the solidified CRA. Plates were incubated at 37 °C for 24 h. The bacterial isolate was considered as slime producing if black-colored bacterial growth was observed and non-slime producing if red- or pink-colored growth appeared.

#### 3.5.2. Quantitative Screening Using Flat-Bottom 96-Well Microplates

The screening was performed according to the method of Sultan and Nabiel [73]. Overnight culture of each MRSA strain was prepared in TSB supplemented with 1% glucose (TSBG); then 1:100 dilution of these cultures in fresh TSBG was performed. Then 200 µL of each 1:100 diluted culture was dispensed in the wells of microplates. Wells containing 200 µL of TSBG only served as a negative control. Plates were incubated statically at 37 °C for 24 h. Turbidity measurement, biofilm staining and visualization were performed as follows: Turbidity was measured at 600 nm, and then planktonic cells and spent medium were discarded. Plates were washed carefully with phosphate-buffered saline (PBS) three times and then left to dry completely in a laminar air-flow cabinet. The attached biofilm in wells was stained with 200 µL of 0.5% crystal violet solution. Plates were left static for 30 min and then washed with distilled water three times and left to dry completely. For biofilm visualization, 150 µL of absolute ethanol was added to extract the violet color and left for 15 min with shaking; violet color was then measured at 570 nm, using a microplate reader (Biotech, Synergy 2, Winooski, VT, USA). Isolates were classified as strong, moderate or weak at biofilm-forming according to the formula reported by Naves et al. [74]:

(1) BF=AB−CW

where BF is biofilm formation, AB is the OD_570_ of stained bacteria cells attached to the wells and CW is the OD_570_ of stained negative control wells.

If BF ≥ 0.300, the isolate is considered a strong biofilm-forming isolate; BF = 0.200–0.299 means the isolate is moderate at BF-forming, BF of 0.100–0.199 is deemed to be weak, and <0.100 is considered a negative biofilm-forming isolate. Then strong or moderate biofilm-forming strains were selected to perform biofilm-inhibition/disruption assays.

#### 3.5.3. The Anti-Virulence Activity of the Extracts

Inhibition of biofilm formation was examined as follows: the relevant bacterial isolates were allowed to form biofilm under the same conditions described in the biofilm screening section (using TSB medium supplemented with 1% glucose (TSBG) and incubated statically for 24 h at 37 °C), in the presence of sub-MIC of the extracts (proven to be colorless concentrations, to avoid color interference with the assay results) ranging from 0.125 to 0.007 mg mL^−1^. Negative control (TSBG and the same concentrations of the extracts) and positive control (1:100 diluted overnight cultures in TSBG) were included. Plates were incubated at 37 °C for 24 h statically. Biofilm was then stained and visualized, as stated before, using 0.5% crystal violet solution.

Disruption of preformed biofilm was examined as follows: relevant bacterial isolates were allowed to form preformed biofilm under the same stated conditions prior to addition of extracts. After that, spent culture medium and planktonic cells were discarded, followed by the addition of fresh TSBG medium containing the same sub-MIC concentrations of each of the extracts (that were used in the assay of inhibition of biofilm formation). Plates were further incubated at 37 °C for 24 h statically. Negative and positive controls were included, as mentioned before. Biofilm was then stained and visualized as described above, using 0.5% crystal violet solution.

Effect on α-hemolysin activity assay was performed according to Ping et al. [49], with minor modification. In brief, 25 µL of defibrinated rabbit RBCs was added to 875 µL of sterile PBS. Eighteen-hour-old TSB cultures of MRSA strains untreated/treated with sub-MIC of the extracts (0.5 × MIC and 0.25 × MIC) were centrifuged at 1000× *g* for 5 min. Then 100 µL of supernatant was added to the rabbit RBCs suspension, gently mixed and incubated at 37 °C for 15 min. The mixtures were centrifuged at 10,000× *g* for 2 min at 20 °C. The degree of hemolysis was measured at 543 nm, using a microplate reader (Biotech, Synergy 2, Winooski, VT, USA). RBCs treated with 1% Triton-X 100 solution served as positive control and considered 100% hemolysis, while PBS-treated RBCs served as the negative control.

Percent hemolysis was calculated according to the following equation:

(2) $$\%\;\mathrm{Hemolysis}\;=\;\frac{{\mathrm{OD}}_{543}\;\mathrm{of}\;\mathrm{sample}\;-\;{\mathrm{OD}}_{543}\;\mathrm{of}\;\mathrm{negative}\;\mathrm{control}}{{\mathrm{OD}}_{543}\;\mathrm{of}\;\mathrm{positive}\;\mathrm{control}}\times 100$$

The effect of the extracts on the pigmentation of relevant MRSA strains was determined qualitatively [53]. Overnight cultures of these isolates untreated/treated with sub-MIC (0.5 × MIC and 0.25 × MIC) of the extracts in TSB were prepared. Extract-treated/untreated cultures were centrifuged at 10,000× *g* for 5 min, and the pellets were washed twice with PBS and were observed for pigmentation by the naked eye. Overnight cultures of extract-untreated bacteria served as the positive control.

In oxidant susceptibility assays, the bacterial cells used in these assays were prepared as follows: 18-h-old overnight cultures of relevant MRSA strains in TSB untreated/treated with sub-MIC (0.5 × MIC and 0.25 × MIC) of the extracts were centrifuged at 1000× *g* for 5 min. Pellets were washed twice with PBS and then re-suspended in PBS; inoculum was adjusted according to the assay requirement.

In susceptibility to killing by H_2_O_2_: turbidity of PBS bacterial suspension was adjusted to 0.5 McFarland turbidity standard (≈10^8^ CFU mL^−1^). Fresh H_2_O_2_ solution in PBS was added to the bacterial suspension to give a final concentration of 200 mM. The mixture was incubated at 37 °C for 2 h, with shaking at 180 rpm. Viable colonies were enumerated on MHA for both extract-treated and untreated cells at time intervals 0, 30, 60, 90 and 120 min [53].

The quantitative catalase enzyme activity assay was performed according to the method described by Iwase et al. [75], but with some modifications. OD_600_ of bacterial suspension in PBS was adjusted to 5. Then 100 µL of the bacterial suspension was added to glass Wasserman tubes (1 cm diameter, 7.5 cm height) containing 100 µL of fresh 30% H_2_O_2_ and 100 µL of 1% Triton-X 100, mixed well and incubated at room temperature for 15 min. The height of the observed O_2_ froth that remained constant for 15 min was measured by using a ruler. The inhibition of enzyme activity was calculated according to the following equation:

(3) $$\%\;\mathrm{Inhibition}\;=\frac{\mathrm{Height}\;\mathrm{of}\;\mathrm{froth}\;\mathrm{of}\;\mathrm{untreated}\;\mathrm{sample}\;-\mathrm{Height}\;\mathrm{of}\;\mathrm{froth}\;\mathrm{of}\;\mathrm{treated}\;\mathrm{sample}}{\mathrm{Height}\;\mathrm{of}\;\mathrm{froth}\;\mathrm{of}\;\mathrm{untreated}\;\mathrm{sample}}\times 100$$

### 3.6. Untargeted Profiling of Different Meliaceae Species by UHPLC-QTOF Mass Spectrometry

The three Meliaceae extracts were re-suspended in dichloromethane solution and centrifuged at 8000× *g* for 10 min. The supernatants were then collected in the LC–MS vials, and then 6 µL of each sample was injected in an ultrahigh-performance liquid chromatography–quadrupole time-of-flight–mass spectrometry (UHPLC–QTOF–MS). A 1290 liquid chromatography coupled with a G6550 mass spectrometer detector via a Dual Electrospray Jet Stream ionization system (Agilent Technologies, Santa Clara, CA, USA) was used. The instrumental conditions for analyzing plant extracts were optimized in previous works [76,77]. The mass spectrometer acquisition was made in the *m*/*z* range 100–1200, using a positive full-scan mode with a nominal resolution at 30,000 FWHM. The Agilent software Profinder B.06 was used to align and annotate the raw mass features according to the “find-by-formula” algorithm, using the combination of monoisotopic accurate mass and the entire isotopic pattern. To this aim, a custom database built by considering both phenolic compounds (Phenol-Explorer 3.6; http://phenol-explorer.eu/, accessed on 30 September 2021) and limonoids reported in the literature for Meliaceae species was used. The MS data were then subjected to a post-acquisition process, retaining only those compounds putatively annotated within 100% of replications in at least one condition. The approach adopted allowed a Level 2 of compound identification [78]. The annotated compounds were then ascribed into classes and quantified by using single pure-standard compounds analyzed by using the same UHPLC–MS method. The standards prepared (purity > 98%) were representative of the following phenolic subclasses: anthocyanins (cyanidin), flavanols and flavonols (catechin), flavones (luteolin), phenolic acids (ferulic acid), lignans (sesamin), stilbenes (resveratrol), lower-molecular-weight phenolics (tyrosols) and limonoids (azadirachtin B). The results were finally expressed as mg equivalents/g dry matter (DM).

### 3.7. Statistical Analysis

In this work, a one-way analysis of the variance (ANOVA), followed by Tukey’s multiple comparisons and unpaired Student’s *t*-tests (*p*-value < 0.05), was performed when considering the antimicrobial, anticancer and antibiofilm assays, using the software GraphPad Prism 6.01 (GraphPad Software, Inc., San Diego, CA, USA). Moreover, the results of each in vitro assay and semi-quantitative analysis of phytochemicals were analyzed by software PASW Statistics 25.0 (SPSS Inc., Chicago, IL, USA) to investigate significant differences (*p*-value < 0.05, Duncan’s post hoc test). Pearson’s correlation coefficients (*p*-value = 0.01, two-tailed) were also calculated by using the same statistical software. The Agilent software Mass Profiler Professional B.12.06 (from Agilent Technologies, Santa Clara, CA, USA; version B.05.00) was then used to elaborate the untargeted UHPLC–MS data. The raw MS dataset was analyzed according to an unsupervised hierarchical clustering analysis (HCA), based on the fold-change (FC) heat map and supervised orthogonal projections to latent structures discriminant analysis (OPLS-DA) through the software SIMCA 13 (Umetrics, Malmo, Sweden), as previously reported [76]. The software also allowed us to record the goodness-of-fit and goodness-of-prediction parameters of the OPLS-DA model (i.e., R^2^Y and Q^2^Y, respectively). Finally, the variables’ importance in projection (VIP score cutoff > 1) was coupled to a fold-change analysis (FC cutoff > 1.2) to find out the most discriminant marker compounds between the three different Meliaceae species under investigation.

## 4. Conclusions

There is growing evidence that infections mediated by biofilm facilitate the development of chronic infectious diseases caused by inappropriate antibiotic use. A promising solution is to use natural extracts that are rich in bioactive compounds, able to eliminate and able to prevent infections. Accordingly, in this work, we studied some limonoids and phenolics-enriched Meliaceae species as promising sources of therapeutic cytotoxic, antibiofilm and anti-virulence agents. Our findings suggest a possible implication of *A. rohituka* and *M. azedarach* extracts as potential candidates to combat MRSA biofilm infections. However, selective isolation of the major constituents and in-depth in vivo study are still missed in this study. The future scope of our project would be covering an optimization of nano-based formulation of the main constituents followed by preclinical studies in animal models. Notwithstanding, toxicological studies are still needed to further guarantee the cytotoxic potential of *A. rohituka* and *M. azedarach*, including selectivity index towards normal cells.

## Figures and Tables

**Table 1:** Phytochemical composition of the different extracts of Meliaceae species. The results are expressed as mean value (*n* = 3; mg/g dry matter) ± standard deviation. Superscript letters within each column indicate significant differences, as resulted by one-way ANOVA and Duncan’s post hoc test (*p* < 0.05).

Tested Extract	Anthocyanins	Other Flavonoids	Lignans	LMW Phenolics	Phenolic Acids	Stilbenes	Limonoids
*- Toona ciliata*	104.16 ± 2.63 ^c^	12.65 ± 0.65 ^c^	33.91 ± 1.44 ^a^	74.85 ± 1.53 ^b^	7.41 ± 0.85 ^a^	4.59 ± 2.7 ^a^	135.93 ± 13.07 ^a^
*- Melia azedarach*	36.35 ± 5.45 ^b^	3.33 ± 0.29 ^a^	59.25 ± 0.32 ^b^	31.74 ± 2.81 ^a^	16.68 ± 0.5 ^b^	3.15 ± 0.25 ^a^	420.63 ± 10.8 ^b^
*- Aphanamixis polystachya*	19.19 ± 1.35 ^a^	7.35 ± 0.59 ^b^	188.56 ± 8.1 ^c^	248.05 ± 8.32 ^c^	6.61 ± 0.56 ^a^	15.1 ± 0.1 ^b^	433.17 ± 12.63 ^b^

## Data Availability

Not applicable.

## Supplementary Materials

The following supporting information can be downloaded online, Figure S1: Hierarchical unsupervised clustering analysis heat map built by considering the phytochemical profile of the three different extracts of Meliaceae species under investigation. Figure S2: Average MIC values recorded for the tested extracts of *Aphanamixis polystachya* and *Melia azedarach* in mg mL^−1^ against five clinical isolates of Methicillin-resistant *Staphylococcus aureus* (MRSA); the results represent the mean of three replicates ± SD. Vancomycin showed average MIC values of 0.002 ± 0.0 mg.mL^−1^ with the 5 tested MRSA strains. Unpaired *t*-test, *p*-value < 0.0001. Figure S3: Inhibition of pigmentation of MRSA-3 and MRSA-5 by extracts of *Aphanamixis polystachya* was more pronounced than the effect of *Melia azedarach* at concentration of either 0.5 × MIC or 0.25 × MIC: (A) 0.5 × MIC treated *A. polystachya* cells, (B) 0.25 × MIC treated *A. polystachya* cells, (C) Extract-untreated cells, (D) 0.5 × MIC treated *M. azedarach* cells and (E) 0.25 × MIC treated *M. azedarach* cells. Table S1: Metabolomic dataset containing the annotated compounds from UHPLC–QTOF–mass spectrometry. Each compound is provided with its relative abundance and composite mass spectrum. Table S2: A comprehensive list of potential VIP marker compounds, together with their VIP score (>1) and LogFC values obtained by comparing the significant metabolites of the 3 different Meliaceae species. Table S3: Pearson’s correlation coefficients regarding the different in vitro biological assays and the annotated phytochemical classes by untargeted metabolomics.
